# Evaluation of Reference Genes for RT-qPCR Studies in the Seagrass *Zostera muelleri* Exposed to Light Limitation

**DOI:** 10.1038/srep17051

**Published:** 2015-11-23

**Authors:** M. Schliep, M. Pernice, S. Sinutok, C. V. Bryant, P. H. York, M. A. Rasheed, P. J. Ralph

**Affiliations:** 1 Plant Functional Biology and Climate Change Cluster (C3), University of Technology Sydney, 15 Broadway, Ultimo, 2007, NSW, Australia; 2 TropWATER - Centre for Tropical Water and Aquatic Ecosystem Research, James Cook University, 1-88 McGregor Road, Smithfield, 4878, QLD, Australia

## Abstract

Seagrass meadows are threatened by coastal development and global change. In the face of these pressures, molecular techniques such as reverse transcription quantitative real-time PCR (RT-qPCR) have great potential to improve management of these ecosystems by allowing early detection of chronic stress. In RT-qPCR, the expression levels of target genes are estimated on the basis of reference genes, in order to control for RNA variations. Although determination of suitable reference genes is critical for RT-qPCR studies, reports on the evaluation of reference genes are still absent for the major Australian species *Zostera muelleri* subsp. *capricorni* (*Z. muelleri*). Here, we used three different software (geNorm, NormFinder and Bestkeeper) to evaluate ten widely used reference genes according to their expression stability in *Z. muelleri* exposed to light limitation. We then combined results from different software and used a consensus rank of four best reference genes to validate regulation in Photosystem I reaction center subunit IV B and Heat Stress Transcription factor A- gene expression in *Z. muelleri* under light limitation. This study provides the first comprehensive list of reference genes in *Z. muelleri* and demonstrates RT-qPCR as an effective tool to identify early responses to light limitation in seagrass.

Seagrasses are considered marine key stone species because of their contribution to a balanced coastal ecosystem^1^,^2^. The wider family of seagrasses (Order: Alismatales) consists of about 60 mostly marine species, which provide a range of extremely important ecosystem services including: acting as nurseries for juvenile commercial fish^3^, prevention of coastal erosion^4^, and they are extremely effective in long-term carbon sequestration (blue carbon). In fact seagrasses are considered one of the most powerful carbon sinks on the planet^5^,^6^,^7^; they are also key components in the nutrient and biogeochemical cycling within coastal habitats^8^,^9^,^10^.

Worldwide, seagrass meadows are declining at an estimated rate of >7% p.a.^2^ due to a range of factors including eutrophication and degrading water quality from coastal development^11^,^12^. Seagrasses also face increasing threats in the future from climate change in the form of amplified storms, sea level rise and elevated seawater temperatures (e.g.^13^,^14^,^15^,^16^,^17^). Seagrasses are highly light dependant requiring far greater levels of irradiance than other marine macrophytes^18^ and therefore light is often the limiting factor affecting seagrass growth and survival^19^,^20^,^21^. Light levels available to seagrasses can be reduced by nutrient-driven algal blooms and increased epiphyte growth on leaf blades^22^, increased turbidity from industrial, urban and agricultural run-off 23, and sediment resuspension from severe weather events that are predicted to be more frequent under future climate change conditions^24^,^25^,^26^. Dredging and port development has also been identified as a major threatening process to seagrasses both globally and within Australia^25^,^27^. While dredging can affect seagrasses by direct removal of plant materials within the dredge footprint or by burial under spoil material, in many instances indirect effects of reduced light from turbid water in sediment plumes has the greatest impact on seagrasses^28^.

Recently, it was determined that seagrass acclimated to low-light conditions were less resilient to an increased low light condition^29^. This finding highlights the importance of knowing the light stress history of a seagrass meadow where a large dredging plume is to be expected in order to make informed management decisions about the likely impact. A molecular toolkit such as a gene expression level indicator, capable of analysing the stress level in a seagrass e.g. as a result of a prolonged low-light period would be very valuable in that respect. A critical point for evaluating stress response reactions in seagrass is to be able to distinguish between both phenotypic plasticity and acclimation or possible adaptation effects^30^,^31^. Having the means to quantify the expression of stress-inducible genes in coastal organisms is highly relevant to marine molecular ecology^32^, as well as for coastal ecosystem management decisions. A molecular toolkit to identify light limitation in seagrass could be a valuable tool to quantify the prevailing light conditions and therefore (i) to identify if plants are under light limitation before a reduction in density takes place and (ii) to determine how vulnerable a meadow would be to increased/prolonged light attenuation e.g. through stress associated with a dredging plume. Having such a tool could greatly benefit future seagrass management and conservation decisions^33^.

The real-time quantitative polymerase chain reaction (RT-qPCR) method is a technique of choice to quantify the expression of stress-inducible genes, being established in various organisms ranging from microorganisms such as bacteria or microalgae to fungi, plants and animals^34^,^35^, from the single cell level^36^ to tissue specific or on whole organism RNA extracts. Even today, with RNA sequencing (transcriptomics) becoming increasingly affordable and hence more widely used, RT-qPCR remains the most reliable and accurate technique for gene expression studies, especially in non-model organisms^37^. The major advantages of RT-qPCR over transcriptomics are that it is highly quantitative, sensitive over a wide dynamic range, and fairly easy to run plus it has a short turn-around time from sample to results^33^,^38^. In order to obtain reliable gene expression level data via RT-qPCR, it is critical to normalize the levels of target genes on the basis of reference genes that are known to be continuously expressed at the same levels, to control for the amount and quality of starting material, enzymatic efficiencies, and overall transcriptional activity. RT-qPCR studies using inappropriate normalization strategies can lead to up to 20 fold error values, which highlights the importance of using several thoroughly tested reference genes^39^. Unfortunately, even today, many studies are using non-validated reference genes throughout all the experimental conditions used. To address this still quite common scientific ‘oversight’ Bustin and co-workers published MIQE (Minimum Information for Publication of Quantitative Real-Time PCR Experiments) guidelines^40^ that are now well-accepted with those guidelines being cited over 3500 times to date (July 2015).

The first RT-qPCR work in seagrasses was performed by Ransbotyn and Reusch^30^, they evaluated 12 candidate reference genes under elevated temperatures regimes in the northern hemisphere seagrass species *Zostera marina*. Since then several other groups in Europe have published selected RT-qPCR data on the two seagrasses *Posidonia oceanica*^41^,^42^,^43^ and *Z. marina*^15^,^44^. However, no validated normalization strategy for RT-qPCR has been published so far in southern hemisphere seagrasses, including *Zostera muelleri* subsp. *capricorni* (*Z. muelleri*)^45^, a species that is ecologically important in Australia and New Zealand, and is presently threatened by coastal development, eutrophication and sedimentation. The aims of the present study were therefore (i) to identify and evaluate a set of reference genes and (ii) to provide a validated normalization strategy for early detection of light limitation in *Z. muelleri* growing in Gladstone Harbour (Queensland, Australia) using RT-qPCR.

## Material and Methods

### Experimental setup and sampling regime

**Figure 1:**
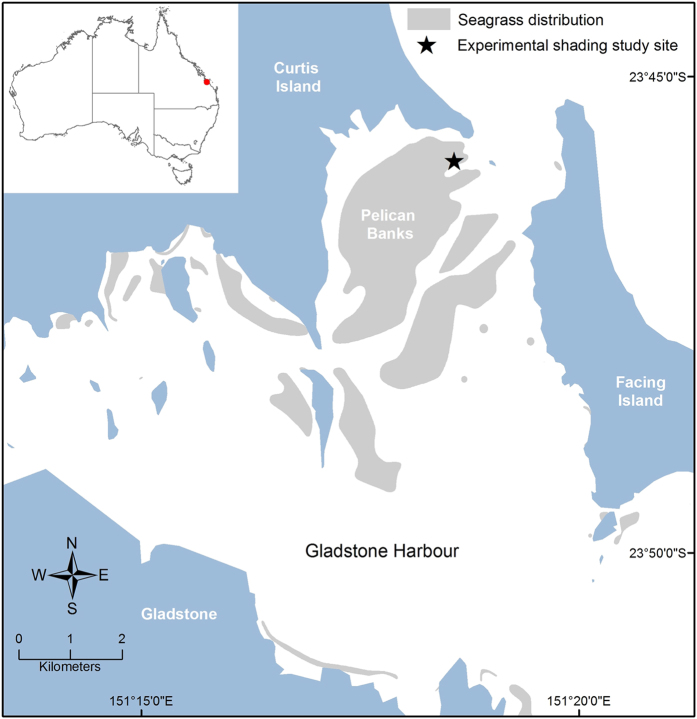
Seagrass distribution map in Gladstone Harbour (Queensland, Australia) and location of the experimental shading study site (map was created using ArcGIS Ver. 10.1; https://www.arcgis.com).

An experimental shading study was established within a relatively homogenous section (approximately 20 m × 10 m) of a large intertidal *Zostera muelleri* spp. *capricorni* meadow at Pelican Banks in Gladstone Harbour (Latitude: 23°46′4.36″S and longitude: 151°18′13.43″E; Queensland, Australia) (Fig. 1). Shade screens were used to assess the impact of a reduction in light on seagrass health and to field test possible candidate and reference genes for the development of a molecular assay to monitor light limitation. A semi-diurnal tide cycle meant that seagrasses were low tide air exposed at least fortnightly. The study was conducted in September 2013 over the peak growth period for seagrasses in the region^20^.

**Figure 2:**
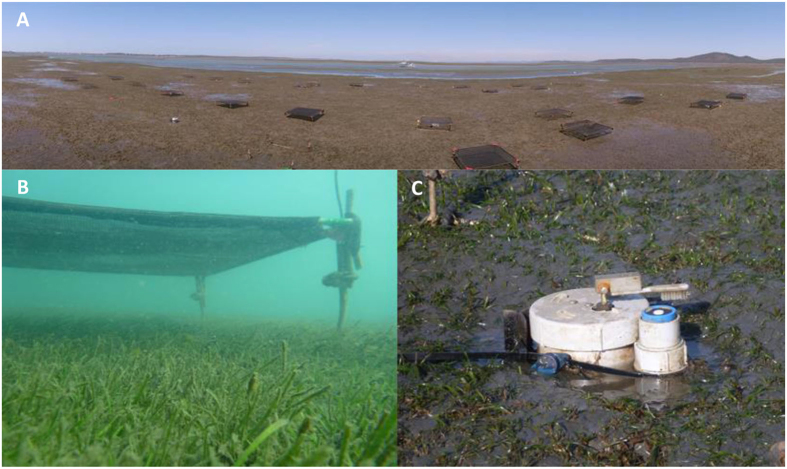
Experimental study site set up. (**A**) Experimental shading study site set up during an exposure event; (**B**) individual treatment plot during submersion; (**C**) irradiance logger and automated wiping apparatus deployed at the experimental shading site (all photographs were taken by and are courtesy of the TropWATER group, James Cook University).

Plots with the shade treatment and control plots were randomly distributed across the study site and neutral density polyethelene shade screens (1 m^2^) were attached at a height of 15 cm above the sediment (Fig. 2A) to create the light attenuation. Control plots were identical to treatment plots but with no shade screen attached. Treatment plots were shaded for a period of 2 weeks (4^th^ to the 18^th^ of September 2013).

During tidal exposure events at 0 weeks and 2 weeks, 3 samples of *Z. muelleri* were randomly collected from both control and treatment plots.). Although pooling samples from different plants and tissues can decrease the stability of gene expression^46^, pools of 2–4 plants (including leaf, meristem, rhizome and root tissues) were used for this experiment to ensure sufficient biomass and RNA yield for each sample. Seagrasses were harvested towards the centre of the plot to control for any edge effects created by the isolation border. Samples were packed in aluminium foil envelopes and placed immediately into liquid nitrogen for transport to the University of Technology Sydney laboratory.

### RNA extraction and sample preparation for RT-qPCR analysis

Whole plant samples were ground into powder using a mortar and pestle in liquid nitrogen. RNA was then extracted using the RNAeasy kit (Life Technologies). Column purification and on-column DNAse I (Life Technologies) digestion was carried out to remove any impurities from the RNA. The RNA quality was assessed using RNA 6000 Nano LabChip Kit (Agilent 2100 Bioanalyzer plant RNA assay, Agilent Technologies, Australia). Good quality RNA samples, with preserved 18 and 28S rRNA bands and RNA integrity number (RIN) >5 were stored at −80° C for further RT-qPCR experiments.

### RT-qPCR experiments

The present study conforms to the Minimum Information for Publication of Quantitative Real-Time PCR guidelines^40^. In this section, we indicate the essential information, *sensu*^40^, required to allow reliable interpretation of the corresponding RT-qPCR results.

### Primer design and RNA extraction

Homologs of the twelve candidates genes (10 reference genes and 2 target genes; Table 1) were identified within the Expressed Sequence Tags (EST) database of *Zostera marina* 47. Six candidate reference genes used in this study (genes coding for 18S ribosomal protein, Actin, Glyceraldehyde 3-phosphate dehydrogenase, Tubulin beta-1 chain, Translation initiation factor 1 subunit beta and Translation initiation factor 2 subunit beta, respectively) were selected based on previous gene expression studies focussing on other seagrass species^30^,^43^. Moreover, four other candidate reference genes were selected since they are involved in ubiquitous cellular processes (genes coding for 30S ribosomal protein S4, Adenosylhomocysteinase, Poly(A) RNA polymerase and Calmodulin, respectively) and therefore widely assumed to be constitutively expressed^48^,^49^,^50^. The EST sequences were used as templates to design sequence-specific primer pairs using the freely available Primer3+ 0.4.0 software package with default settings^51^. The sizes of the resulting amplicons were kept in a narrow size range from 79 to 197 bp ensuring similar PCR efficiencies and facilitating cross-comparison of assays. The specificity of each selected primer pair was observed by PCR amplification as single bands at the expected size resolved via agarose gel electrophoresis (data not shown).

### Differential gene expression analysis

Efficiency of target gene amplification was optimised prior to running samples for each primer pair by trialling 2 primer concentrations (500 and 100 nM). The cDNA was generated from the extracted RNA with a High Capacity Reverse Transcription kit (Applied Biosystems). Triplicate cDNA aliquots from each sample served as templates for RT-qPCR using SYBR Green PCR Master Mix (Applied Biosystems) on a Step One Plus Real-Time PCR System (Applied Biosystems). Amplification of 10 μL reactions with 0.5 ng of cDNA from treated and control seagrass samples, and 100 nM of each specific primers were placed in 96-well optical plates with the following PCR conditions: incubation at 94 °C for 10 min, then 40 cycles of 94 °C, 60 °C, 68 °C for 30 sec each followed by 68 °C for 5 min. The RT-qPCR efficiency for each gene and each treatment was determined from a cDNA dilution gradient of 27, 9 3 and 1 ng and a linear regression model^52^. The corresponding RT-qPCR efficiencies were calculated according to the equation below^49^:


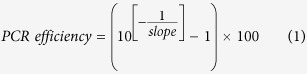


While the usual practice consists of accepting a pair of primers as valid if its efficiency is above 90%, pair of primers have been accepted previously with efficiency as low as 85%^53^. In this study, all the RT-qPCR efficiencies obtained with the different primers were comprised between 88–114%, with a calibration coefficients >0.95 (Table 1 and Supplementary Figure S1). A *no template control*, as well as a *no reverse transcription control* was generated for each gene and each treatment to ensure that the PCR reactions were free of DNA contamination.

### Data acquisition

Data from RT-qPCR was analysed using the Step One Plus Software (Ver. 2.3; Applied Biosystems). Expression levels were determined as the number of cycles needed for the amplification to reach a fixed threshold in the exponential phase of the RT-qPCR reaction^54^. The cycle threshold (C_T_) was set at 0.03 for all genes. The corresponding C_T_ values were used directly in the software packages geNorm^55^, NormFinder^56^ and Bestkeeper^57^ to rank and select the most stable reference genes. To validate changes in target genes expression, C_T_ were imported into the qbase+ software package (Biogazelle) and transformed into quantities using maximum efficiency of 1.00 (or 100%) to obtain Calibrated Normalized Relative Quantities (CNRQ)^58^.

### Selection of reference genes

In order to select the best reference genes for the experimental conditions, expression stability was analysed using BestKeeper, geNorm, and NormFinder according to their established protocols.

### BestKeeper

BestKeeper was used to determine standard deviation and power of each reference gene, and then to select the best reference genes based on these variables as described in^57^.

### GeNorm

GeNorm was used (i) to rank the best reference genes based on their M stability (M) and to determine the optimal number of reference genes to be used based on the Pairwise variation (V) as recommended by^55^.

### NormFinder

NormFinder was used to provide a stability value for each candidate gene (i.e. direct measure for the estimated expression variation) as previously described by^56^.

### Consensus ranks of candidate reference genes

We established a consensus rank of genes by combining stability measurements produced by geNorm, NormFinder and BestKeeper by using the RankAggreg package (http://cran.rproject.org/web/packages/RankAggreg/index.html)^59^. Briefly, we imported the matrix of rank-ordered genes according to the 3 different software packages to the RankAggreg statistical package, calculated the “distance” between ordered lists using the Spearman foot rule function and performed rank aggregation via the Cross-Entropy Monte Carlo algorithm. The consensus ranking with the lowest score was then chosen.

### Statistical analysis

Statistical analyses were done using the software Statistica 7.0 (Statsoft Inc., Tulsa, OK, USA). Kolmogorov-Smirnov and Levene’ test were used to first assess the data for normality and homoscedasticity, respectively. The data was found to be normal and homogenous, and therefore parametric test (t-test) was used to test the effect of light limitation on the expression level of target genes. Throughout this paper, values given are the mean of 3 biological replicates (each biological replicate being a sample pool of 2–4 plants each), including technical triplicates for RT-qPCR data. Results were considered significant at 5%.

## Results

All the tested reference genes showed a high level of expression with C_T_ value below 35, as recommended previously^60^, and were selected for further evaluation of expression stability. As target genes, we used Photosystem I reaction center subunit IV chloroplast precursor (PSI)^44^ and Heat Stress Transcription factor A-1d (HSF)^61^.

The RT-qPCR efficiencies were similar between the two concentrations for the 12 candidate genes (10 reference genes and 2 target genes) and we selected the lowest concentration (i.e. 100 nM) for all primer pairs. Dissociation-curve analysis after 40 cycles of amplification revealed that all primer pairs amplified a single PCR product and gel electrophoresis of the PCR products confirmed the expected amplicon sizes (data not shown). All PCRs displayed coefficients of correlation greater than 0.95 and efficiencies ranged between 88 and 114% (Table 1 and Supplementary Figure S1). Replicate variability of the C_T_ values between the 3 technical replicates was examined for each sample-gene combination. Repeatability of the assay between the technical replicates was consistent across the different genes with the replicate variability falling within the set limit of <0.5 cycles for all the sample-gene combinations tested.

**Figure 3:**
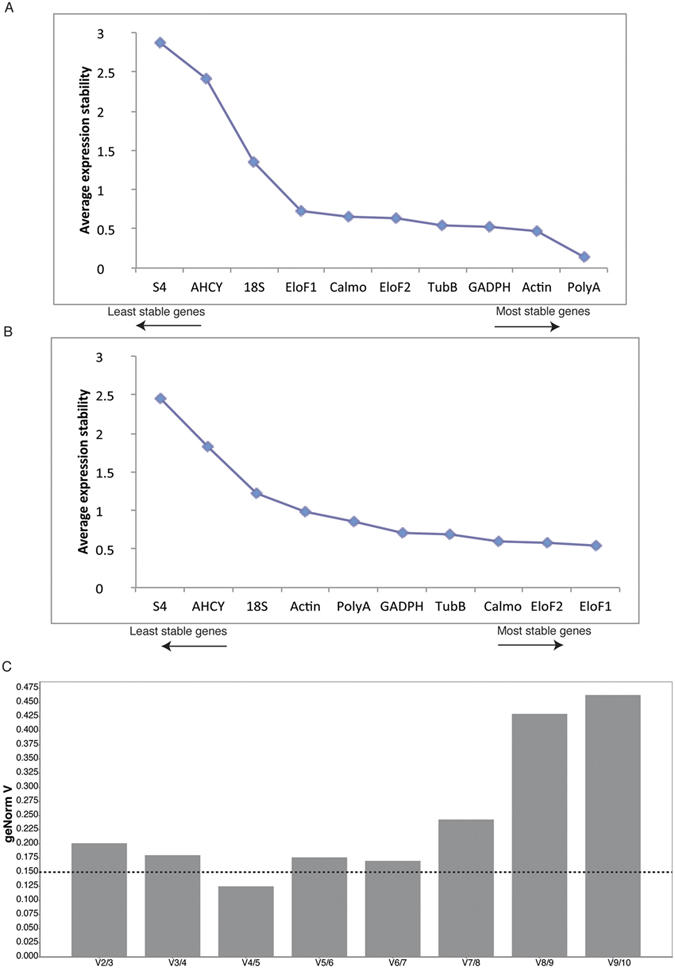
Analysis of expression stability of 10 potential reference genes using Normfinder and geNorm software in *Zostera muelleri* exposed to light limitation. Average expression stability value (as defined by Normfinder, **A** and by geNorm, **B**) for each candidate gene and pairwise variations (as defined by geNorm, **C**) for determination of optimal number of reference genes for normalization. The dashed line indicates 0.15 as the cut-off value below which the inclusion of an additional reference gene is not needed. S4: 30S ribosomal protein S4; AHCY: Adenosylhomocysteinase; 18S: 18S ribosomal protein; PolyA: Poly(A) RNA polymerase; GADPH: Glyceraldehyde 3-phosphate dehydrogenase; TubB: Tubulin beta-1 chain; Calmo: Calmodulin; EloF1b: Translation initiation factor 1 subunit beta; EloF2b: Translation initiation factor 2 subunit beta.

Selection and validation of the best references genes for accurate normalization of gene expression under light limitation was conducted using geNorm software^49^ within the qbase+ software package, BestKeeper^57^ and NormFinder^56^. All samples were measured in the same run for a given reference gene (i.e. sample maximization strategy^58^). Reference gene expression stability was defined by (i) M (average expression stability, lower M values corresponding to more stable gene expression) in NormFinder and geNorm software (Fig. 3A,B). For most of the reference genes, the M values were under 1, reflecting a relative stability in response to light attenuation, except for S4 and adenosylhomocysteinase genes (M value between 1 and 3 for NormFinder, Fig. 3A and M value between 1 and 2.5 for geNorm, Fig. 3B). Still, the candidate reference genes displayed a wide range in their M values, the most stable expression under light limitation was found for PolyA for NormFinder (M = 0.14, Fig. 3A) and translation initiation factor 1 subunit beta for geNorm (M = 0.545; Fig. 3B). Pairwise variation (V) analysis was then used to determine the optimal number of reference genes to be used (i.e. by estimating the effect of including an additional gene^55^. Vandesompele *et al.*^55^ proposed pairwise variation of 0.15 as a flexible cut-off value below which the inclusion of an additional reference gene is not needed. According to this cut-off value, our results indicate that the optimal number of reference genes to accurately normalise expression of target genes in *Z. muelleri* in response to light limitation was 4 (V4/5 = 0.115, Fig. 3C). The addition of a fifth reference gene did not result in a further decrease of the normalisation factor, but a rather significant increase (V5/6 = 0.174, Fig. 3C).

**Figure 4:**
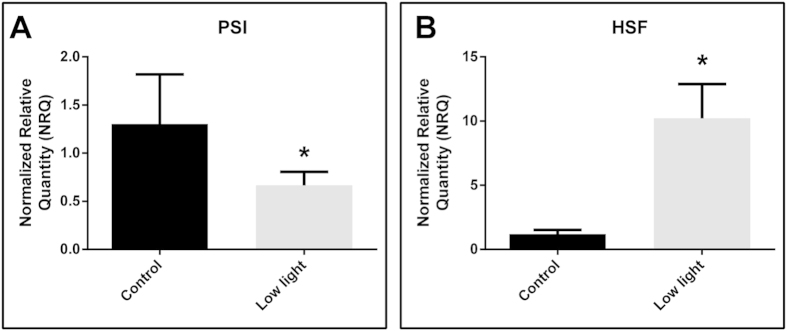
Effect of 2 weeks of shading on gene expression in *Zostera muelleri*. Relative expression of PSI (Photosystem I, reaction center subunit IV, chloroplast precursor, (**A**) and HSF (Heat Stress Transcription factor A-1d, (**B**) were normalized to the consensus four most stable reference genes (Poly(A) RNA polymerase, Glyceraldehyde 3-phosphate dehydrogenase, Translation initiation factor 1 subunit beta and Tubulin beta-1 chain). The statistical difference between means is indicated as *P < 0.05. Error bars represent SEM with 3 biological replicates.

Using BestKeeper, the coefficient of determination of each gene gave the highest reliability to GADPH, PolyA, S4 and TubB (Table 2). A comparison of the rankings produced by the three approaches (geNorm, Normfinder and BestKeeper) indicated significant divergences (Table 3). However, by using the RankAggreg package we were able to establish a consensus list between the 3 different algorithms. This consensus list included PolyA, GADPH, ELOF1 and TubB as the best four reference genes (Table 3), which were then used to evaluate target gene expression profile in *Z. muelleri* under light limitation. The relative quantification^55^ demonstrated that the level of expression of PSI and HSF genes were significantly different under light limitation with the two weeks of shading treatment resulting in a 2-fold reduction of the level of expression for the PSI gene (Fig. 4A, Control = 1.303 ± 0.516; Low light = 0.665 ± 0.140; T-test, t_3_ = 2.295, p = 0.0416) and in an 9-fold up-regulation for the HSF gene (Fig. 4B, Control = 1.196 ± 0.567; Low light = 10.217 ± 4.615; T-test, t_3_ = 2.196, p = 0.0465).

## Discussion

This study is the first to identify stable reference genes for a southern hemisphere seagrass with the results paving the way for the development of molecular toolkits to evaluate the effects of light limitation in *Z. muelleri.*

Based on the pairwise variation analysis, under the experimental low light condition tested, RT-qPCR should ideally be performed using 4 reference genes as internal controls (V value of 0.115). It is generally recommended using between two and five validated reference genes for accurate normalization of RT-qPCR data^55^. Our results support this, showing that the geNorm V value remains in a narrow band of 0.12-0.2 (with 0.15 being the target threshold value) when 2, 3 or 4 of the most stable reference genes are used (Fig. 3C). Therefore, running the experiment with 2 or 3 of the most stable reference genes might be acceptable and will reduce the number of assays to be run (or costs and time) significantly in comparison to using 4.

Having identified the optimal number of reference genes to be used under low light conditions, gene expression analysis of field shading studies will be possible once a range of low light indicator target genes are selected and appropriate primers designed. However, as described in the introduction, the inclusion of additional stressors such as additional light attenuation or nutrient loading in the seawater to simulate eutrophication or even an extended period of the same stress condition might affect: (i) the pairwise variation, resulting in an increase of the optimal number of reference genes needed for normalization of the target gene and (ii) the average expression stability of tested reference genes^48^. Although the reference genes selected in this study appear to be stable expressed when plants are exposed to low-light, we highlight again that for any other RT-qPCR gene expression level analysis experiment in *Z. muelleri*, further investigations to determine stable reference gene expression levels are required, especially if a different stress parameter is being applied.

In the present study, 18S and AHCY genes were consistently ranked among the 3 least stable reference genes under low light conditions for the different algorithms used (Table 3). 18S rRNA gene is a common reference gene^62^, but its use as an internal control for normalisation has been questioned recently due to (i) its great abundance and (ii) its potential imbalance of rRNA and mRNA fractions between different samples, resulting in high variability across different samples/treatments^38^,^40^,^55^,^63^. In contrast, the ranking of the four best reference genes was somehow divergent between the 3 algorithms used (i.e. EloF1, EloF2, Calm, TubB for geNorm; PolyA, Actin, GADPH, TubB for NormFinder and GADPH, PolyA, S4 and TubB for BestKeeper, Table 3), which could be due to differential sensitivity to correlated gene expression patterns as previously observed by Ponton *et al.*^63^. Indeed, the list of candidate reference genes used in this study included two different ribosomal gene (i.e. S4 and 18S) and two different translation initiation factors (i.e. EloF1a and EloF2b) that might be co-regulated. Future studies may provide more convergent results on reference genes in *Z. muelleri* by including only one candidate gene per gene family, therefore potentially decreasing the number of co-regulated genes. By using the RankAggreg package, we were able to combine the stability measurements produced by geNorm, NormFinder and BestKeeper and to establish a consensus list as described by Ponton *et al.*^63^ with PolyA, GADPH, EloF1 and TubB as the best four reference genes (Table 3). Our normalization strategy based on this consensus list of the 4 best ranked reference genes was further evaluated by measuring the expression level of two target genes in *Z. muelleri* when it was exposed to low light.

The PSI target gene is the At2g20260 gene locus (PsaE-2 or Photosystem I reaction center subunit IV B) and corresponds to the homolog of the PsaE-2 gene in *Arabidopsis thaliana*. This stress marker target gene (PSI) was previously selected by Winters *et al.*^44^. PsaE-2 in *A. thaliana* is a membrane-bound, stroma side oriented part of Photosystem I that is thought to assist in the docking of the ferredoxin to Photosystem I and to interact with the ferredoxin-NADP oxidoreductase^64^. Its exact function hasn’t been elucidated to date, but it is believed to play a role in facilitating the cyclic electron transfer of Photosystem I in plants because knock-out mutant experiments revealed that PsaE-2 is not essential for linear electron transport and a functional Photosystem I^64^. Our results demonstrate a significant effect of low light on the level of expression of the PSI gene in *Z. muelleri* with a 2-fold down regulation relative to the control after two-weeks of shading. While it is unclear if this regulation of PSI gene expression reflects stress or acclimation to low-light conditions, it is in agreement with recent gene expression level changes of photosystem subunits genes in *Posidonia oceanica*, a seagrass species endemic of the Mediterranean sea^65^. Indeed, Dattolo *et al.*^65^ studied gene expression in *P. oceanica* along its bathymetric distribution (at −5 m and −25 m) and revealed a 2.3-fold decrease in Photosystem I reaction center subunit V in the deeper stand of the seagrass meadow (25 m deep, low light condition). In congruence with these data and its putative role in cyclic electron transfer of Photosystem I, our results suggest a role of Photosystem I reaction center subunit IV protein in the adaptation to a low-light regime.

The HSF heat stress transcription factor A-1d (with a high sequence similarity to the *Arabidopsis thaliana* AT1G32330 gene locus) is part of the HSFA-1family of HSFs. The exact functions of the HSFA-1s are not fully understood yet but recent research clearly suggests that they are key switches in a range of major HSF responses such as heat and other abiotic stresses in *Arabidopsis*; e.g. as key factors regulating ascorbate peroxidase 2 (APX2) expression^61^. The APX responses are directly involved in the protection of plant cells against adverse environmental conditions^66^.

Although further functional investigations are needed to precisely determine the physiological and molecular mechanisms of PSI and HSF genes in seagrass response to light limitation, the presented data indicates that significant changes in the expression levels of these 2 genes can be detected just 2 weeks after the onset of light limitation, i.e. long before any morphological changes in seagrass can be detected. Morphological changes generally become apparent in seagrasses after 2 months of light limitation^20^,^33^. Our results therefore highlight the potential of these genes to be used as an early marker of light limitation in *Z. muelleri.*

The availability of a set of reference genes for RT-qPCR in *Zostera muelleri* is a critical first step in the development of a molecular assay for reactive monitoring of light limitation in seagrasses in Gladstone Harbour. Given the increasing demand for sub-lethal indicators of stress in response to anthropogenic damage in coastal ecosystems, this knowledge is of significant importance for seagrass management and conservation.

## Additional Information

**How to cite this article**: Schliep, M. *et al.* Evaluation of Reference Genes for RT-qPCR Studies in the Seagrass *Zostera muelleri* Exposed to Light Limitation. *Sci. Rep.* **5**, 17051; doi: 10.1038/srep17051 (2015).

## Supplementary Material

Supplementary Figure S1

## Figures and Tables

**Table 1:** Reference genes and target genes investigated in *Zostera muelleri* by using RT-qPCR.

Name	Accession number	Primer forward sequence	Primer reverse sequence	Length (bp)	Tm	C_T_	Efficiency (%)
S4	Zoma_Contig219	ATGGTCTGACA	TGTTATCCAA	108	59.7	29	114
		GAGCGACAA	ACGCATCTCG				
AHCY	Zoma_C_c69135	TTCTTCATCTT	GCATGGAAGGT	118	60.1	31	113
		GCGCATCTG	CTCCAAGTC				
18S	Zoma_C_c67952	AACGAGACCTC	AAGATTACCCA	189	60.2	26	88
		AGCCTGCTA	AGCCTGTCG				
Actin	Zoma_ZMF02257	TAAGGTCGTTG	ACTCTGCCTTT	104	60.4	26	110
		CTCCTCCTG	GCAATCCAC				
PolyA	Zoma_C_c36619	GCTGCTCGTTC	ATGACCGCCAT	112	59.9	29	93
		AAATTCCTC	TTAATCTGC				
GADPH	Zoma_C_c6252	CGGTTACTGTA	CAAAGGCTGGG	79	59.9	25	88
		GCCCCACTC	ATTGGTTTA				
TubB	Zoma_Contig120	GGACAAATCTT	TCCAGATCCAG	195	60	24	88
		CCGTCCAGA	TTCCACCTC				
Calmo	Zoma_B_i07192	ATCCATCCTGG	CACTGTGATCC	197	60.1	23	114
		TCTTTGTCG	ACTCGTTGG				
EloF1	Zoma_C_c59090	AAGCAAAGGC	TCTGCTGCCTTC	82	59.9	24	104
		GTCACTTGAT	TTCTCCTC				
EloF2	Zoma_B_i03951	GGAAGATTTGC	GCAACAAAAC	174	60	26	102
		ACCCAAGAA	CTGCCTTGAT				
PSI	Zoma_C_c25681	GGGAACCAAG	GAATCTCACCA	121	59.7	35	100
		GTGAAGATT	CAACTGGGTA				
HSF	Zoma_B_i13463	GGATGGACCTTCCTAAATCCA	ACACCTTCCGAGTTTTGCAC	107	60.1	34	95

Accession numbers of the closest sequence matches available online in the data repository for *Zostera marina* EST (http://drzompo.uni-muenster.de/), primers sequences, amplicon length, melting temperature, geometric mean of cycle threshold (C_T_) and RT-qPCR efficiency are indicated. S4: 30S ribosomal protein S4; AHCY: Adenosylhomocysteinase; 18S: 18S ribosomal protein; PolyA: Poly(A) RNA polymerase; GADPH: Glyceraldehyde 3-phosphate dehydrogenase; TubB: Tubulin beta-1 chain; Calmo: Calmodulin; EloF1b: Translation initiation factor 1 subunit beta; EloF2b: Translation initiation factor 2 subunit beta; PSI: Photosystem I, reaction center subunit IV; HSF: Heat Stress Transcription factor A-1d.

**Table 2:** Summary statistics generated by the BestKeeper analysis for candidate reference genes based on cycle threshold (CT).

	GADPH	18S	EloF1	Calmo	EloF2	TubB	Actin	AHCY	PolyA	S4
N	6	6	6	6	6	6	6	6	6	6
Geometric Mean (Ct)	24.29	25.87	24.19	23.10	26.28	24.29	25.92	31.05	28.69	29.17
Standard deviation (±Ct)	0.97	1.55	0.89	0.84	1.06	0.99	0.69	2.76	0.75	4.23
Coefficient of determination	0.686	0.284	0.308	0.355	0.497	0.608	0.456	0.045	0.677	0.638
P value	0.042	0.276	0.254	0.213	0.117	0.067	0.141	0.689	0.044	0.056

**Table 3:** Ranking of candidate reference genes according to their stability value using geNorm, Normfinder and BestKeeper analyses.

GeNorm	NormFinder	BestKeeper	Consensus 3 genes	Consensus 4 genes	Consensus 5 genes	Consensus 6 genes
EloF1	PolyA	GADPH	PolyA	PolyA	GADPH	GADPH
EloF2	Actin	PolyA	GADPH	GADPH	PolyA	PolyA
Calm	GADPH	S4	EloF2	EloF1	EloF2	EloF2
TubB	TubB	TubB		TubB	TubB	TubB
GADPH	EloF2	EloF2			Actin	Calmo
PolyA	Calmo	Actin				Actin
Actin	EloF1	Calmo				
18S	18S	EloF1				
AHCY	AHCY	18S				
S4	S4	AHCY				

Candidates are listed from top to bottom in order of decreasing expression stability.
