# Supplementary material for: Evaluation of Reference Genes for RT-qPCR Studies in the Seagrass Zostera muelleri Exposed to Light Limitation
Source: Sci Rep. 2015 Nov 23;5:17051. doi: 10.1038/srep17051 (PMC4655411; doi:10.1038/srep17051)
Supplement: Supplementary Figure S1 [file srep17051-s1.pdf]

EVALUATION OF REFERENCE GENES FOR RT-QPCR STUDIES IN THE SEAGRASS *ZOSTERA MUELLERI* EXPOSED TO LIGHT LIMITATION

AUTHOR NAMES AND AFFILIATIONS

SCHLIEP, M.[a, *, +], PERNICE, M.[a, *, +], SINUTOK, S. [a], BRYANT, C.V. [b], YORK, P. H. [b], RASHEED, M.A. [b], RALPH, P.J. [a]

[a] Plant Functional Biology and Climate Change Cluster (C3), University of Technology Sydney, 15 Broadway, Ultimo, 2007, NSW, Australia

[b] TropWATER - Centre for Tropical Water and Aquatic Ecosystem Research, James Cook University, 1-88 McGregor Road, Smithfield, 4878, QLD, Australia

[+] Both authors contributed equally.

[*] Corresponding authors

email: martin.schliep@uts.edu.au; telephone: +61 2 9514 4162

email: mathieu.pernice@uts.edu.au; telephone: +61 2 9514 4162

This PDF file includes:

Supplementary Figure S1

Supplementary Figure S1

Supplementary information including standard curves, efficiency and R-square for all the reference and target genes investigated in this study: S4 (A); AHCY (B); 18S (C); Actin (D); PolyA (E); GADPH (F); TubB (G); Calmo (H); EloF1 (I); EloF2 (J); PSI (K); HSF (L).

A

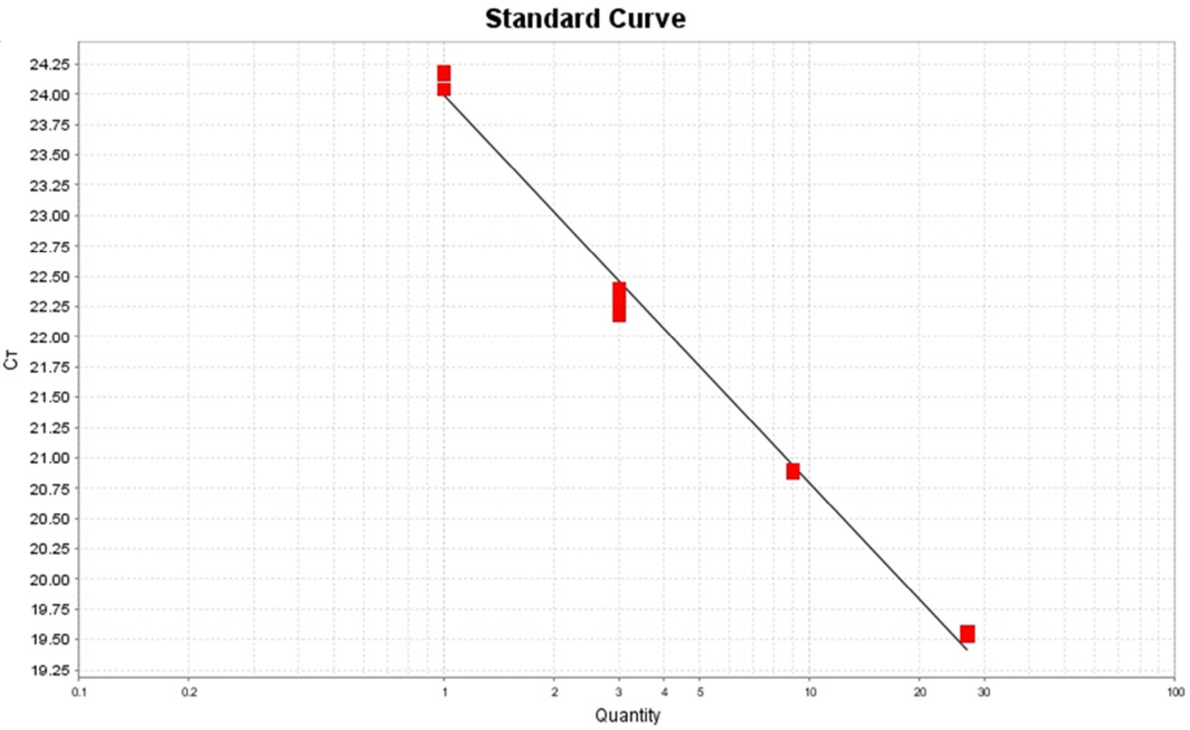

Legend: Standard Target: S4 Slope:-3.011 Y-inter: 30.108 R^2: 0.992 Eff: 114%

B

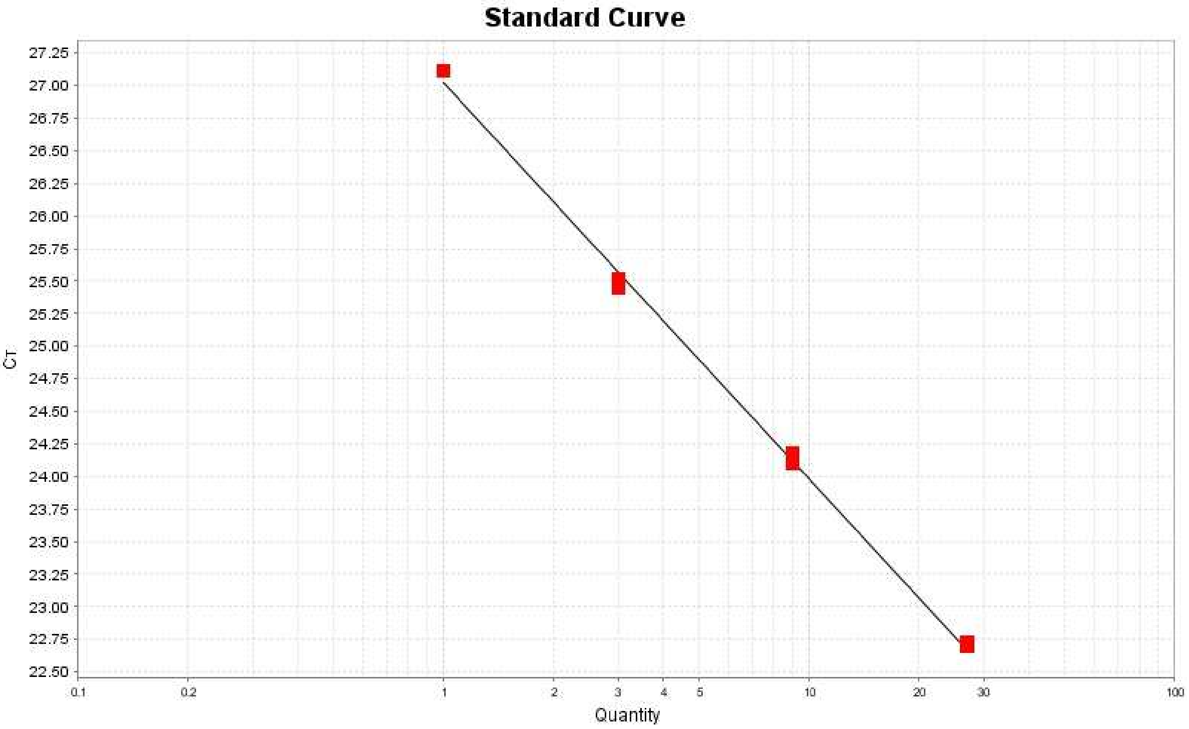

Legend: Standard Target: AHCY Slope:-3.04 Y-inter: 27.025 R^2: 0.97 Eff: 113%

C

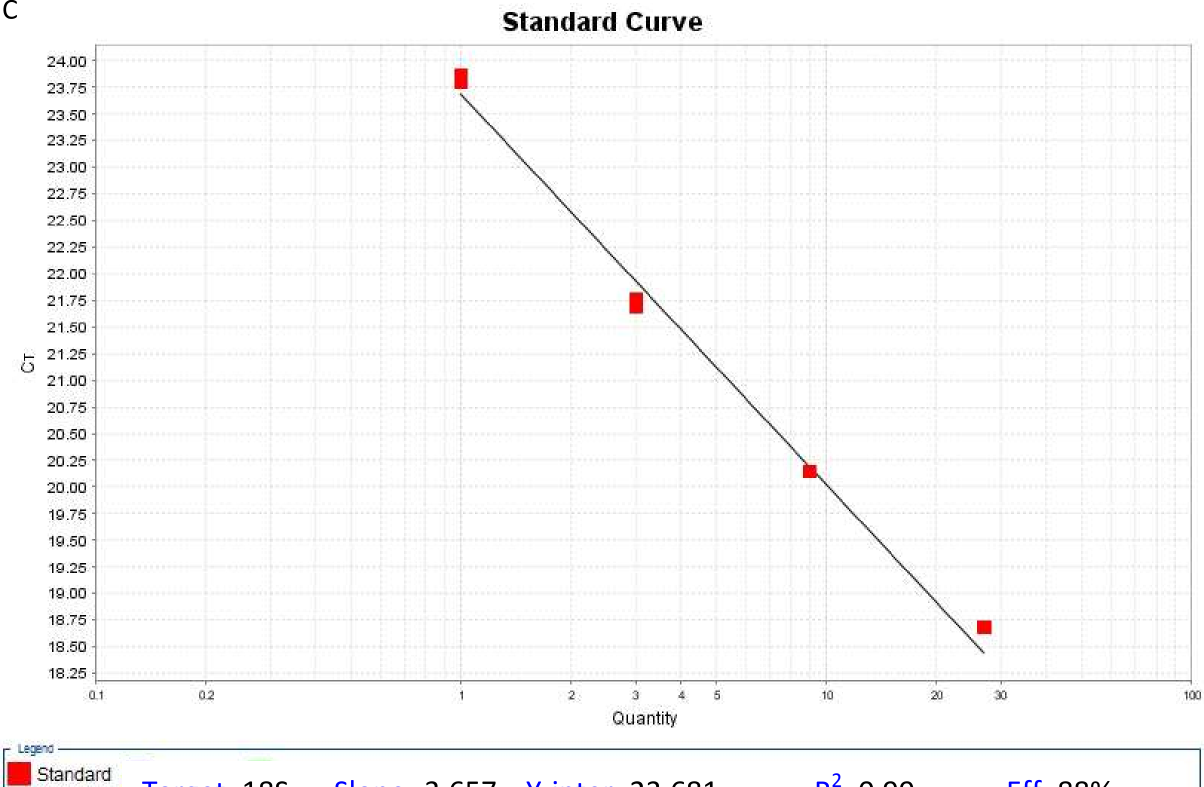

Target: 18S Slope:-3.657 Y-inter: 23.681 R^2: 0.99 Eff: 88%

D

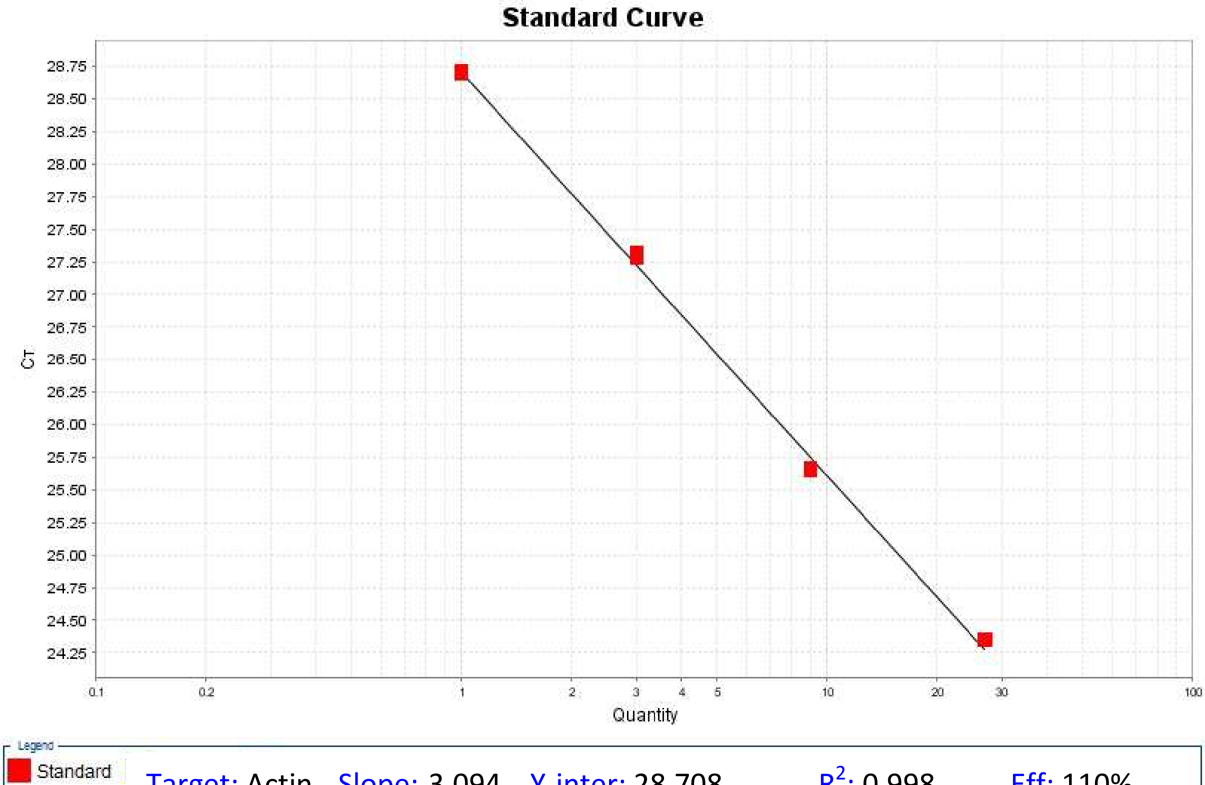

Target: Actin Slope:-3.094 Y-inter: 28.708 R^2: 0.998 Eff: 110%

E

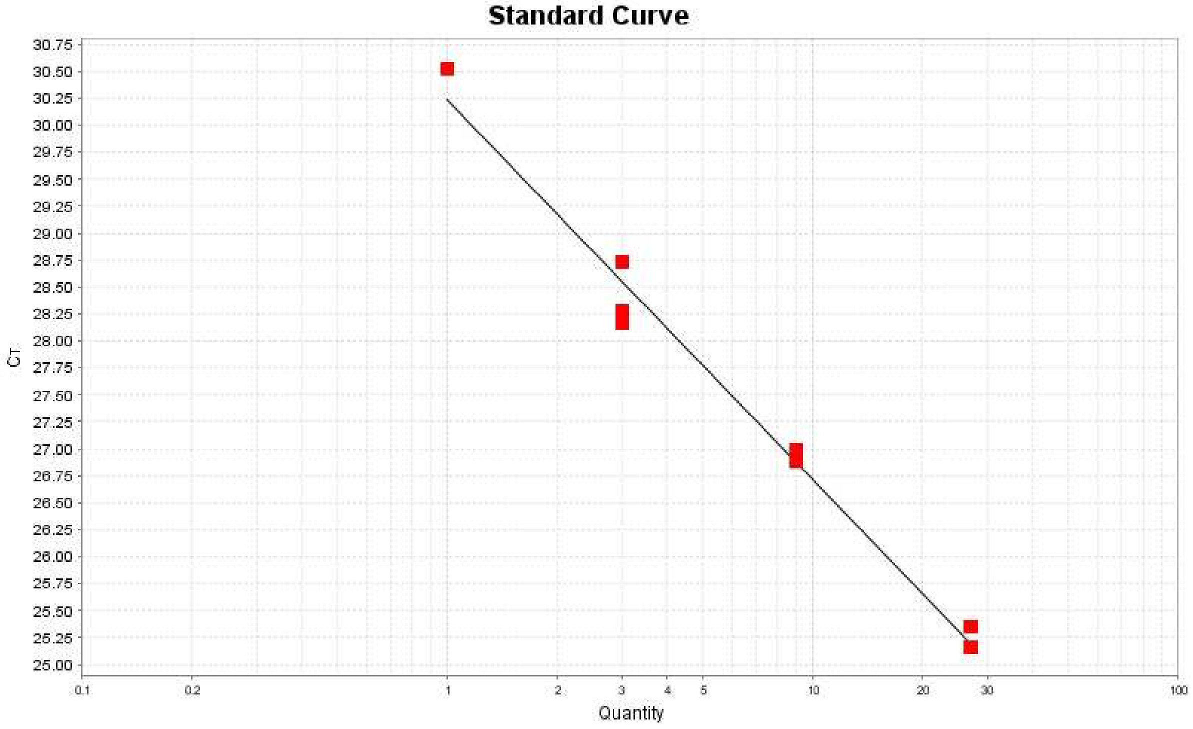

Legend
Standard Target: PolyA Slope:-3.509 Y-inter: 30.229 R^2: 0.983 Eff: 93%

F

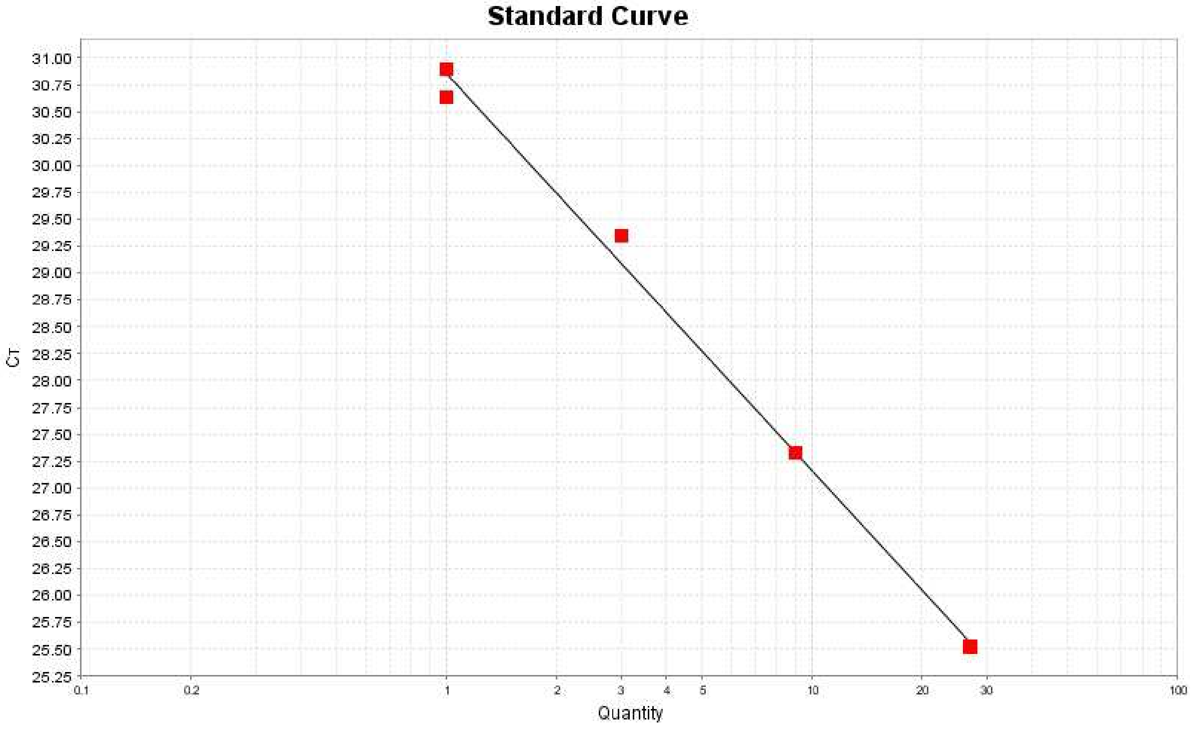

Legend
Standard Target: GADPH Slope:-3.662 Y-inter: 30.816 R^2: 0.994 Eff: 88%

G

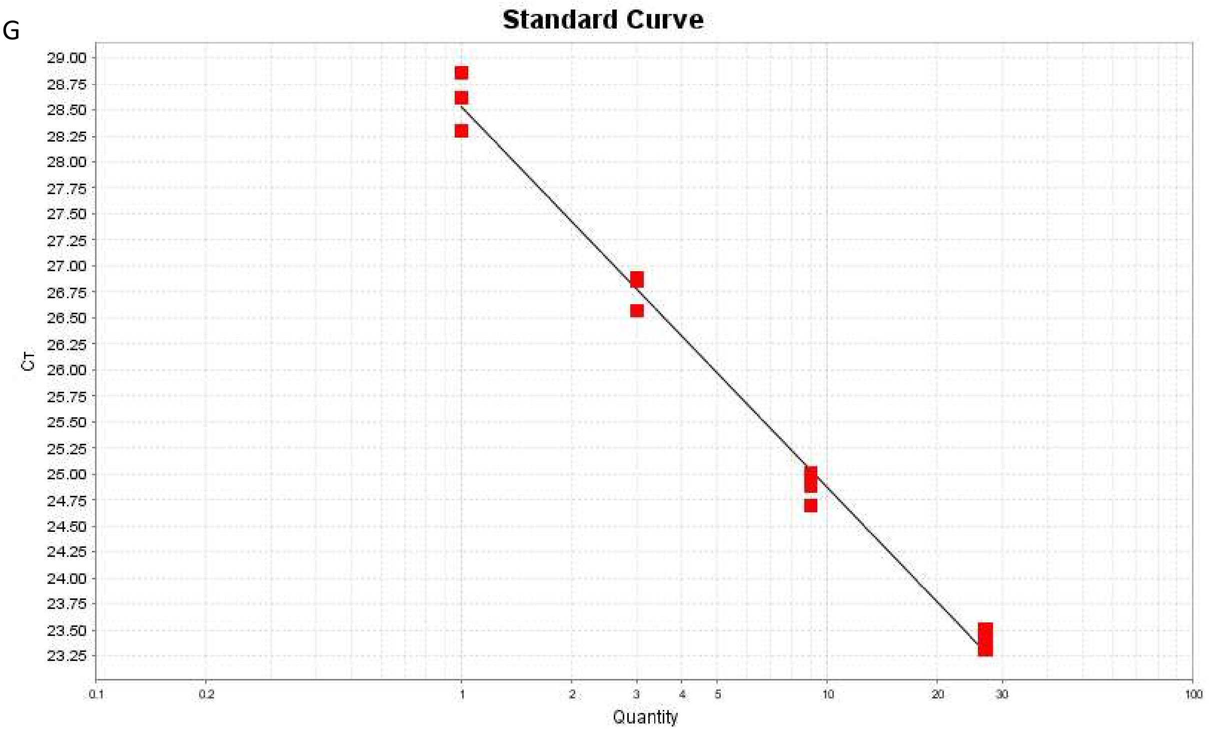

Legend
Standard
Target: TubB Slope:-3.654 Y-inter: 28.527 R^2: 0.99 Eff: 88%

H

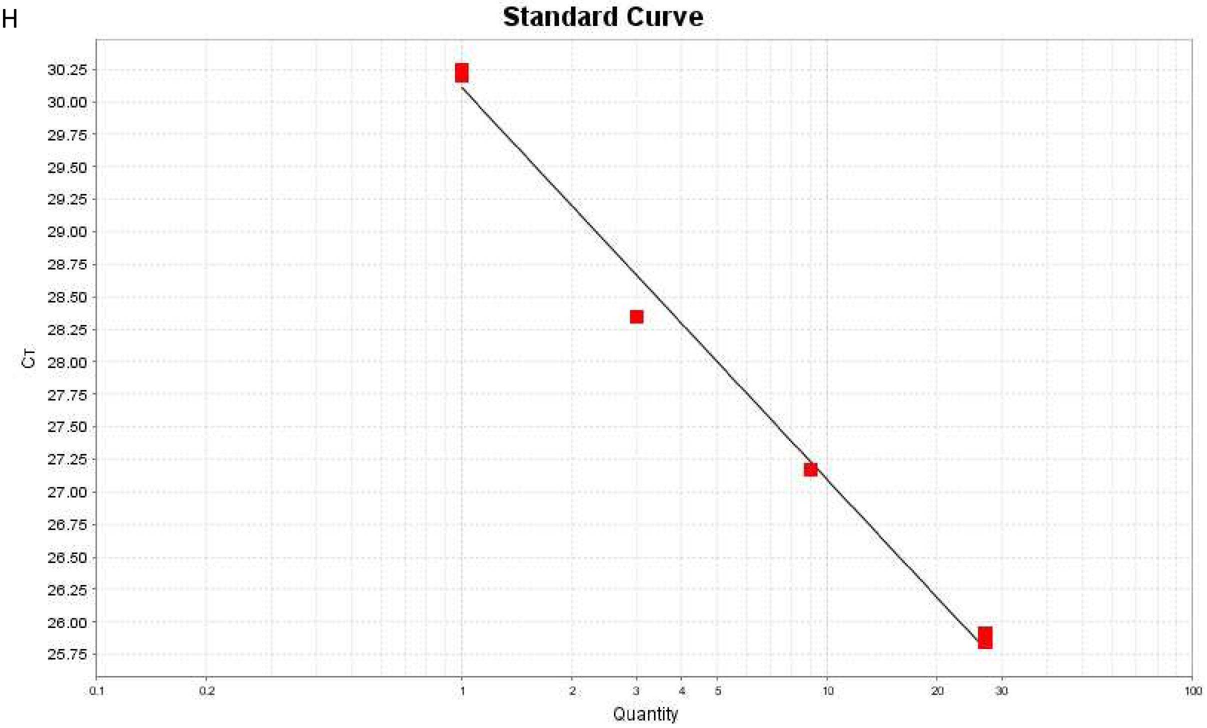

Legend
Standard
Target: Calmo Slope:-3.011 Y-inter: 30.108 R^2: 0.992 Eff: 114%

I

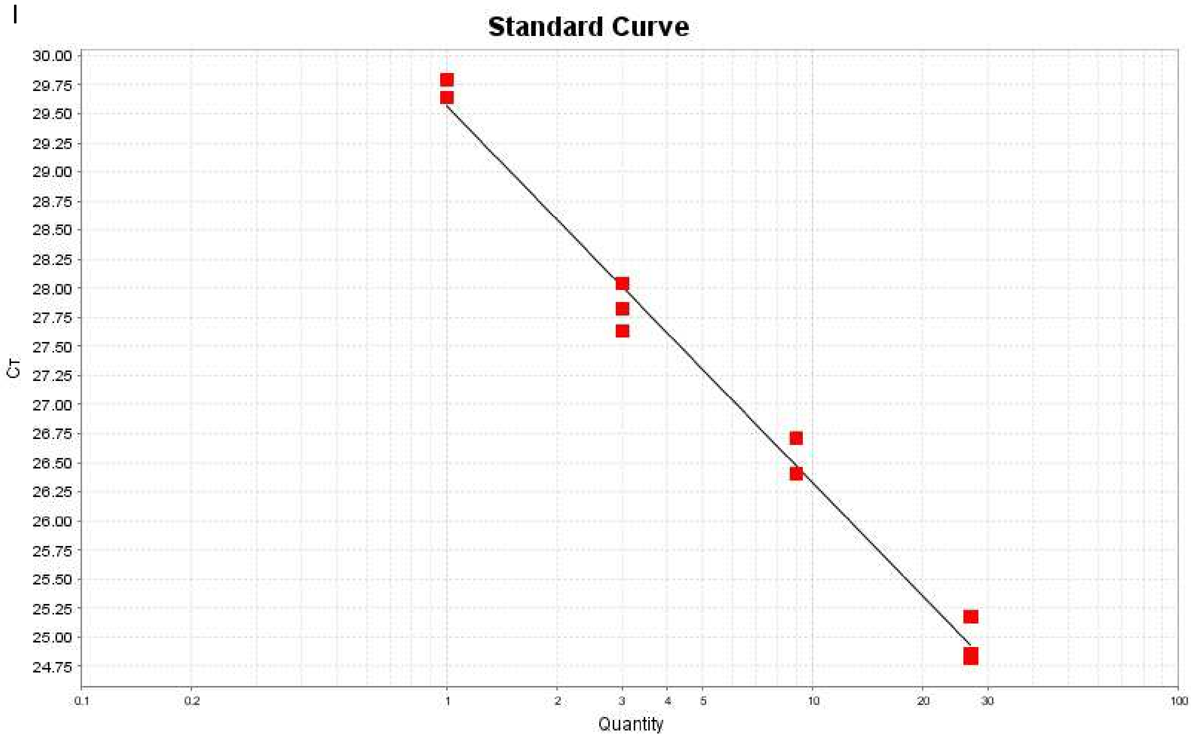

Legend
Standard
Target: EloF1 Slope:-3.237 Y-inter: 29.564 R^2: 0.987 Eff: 104%

J

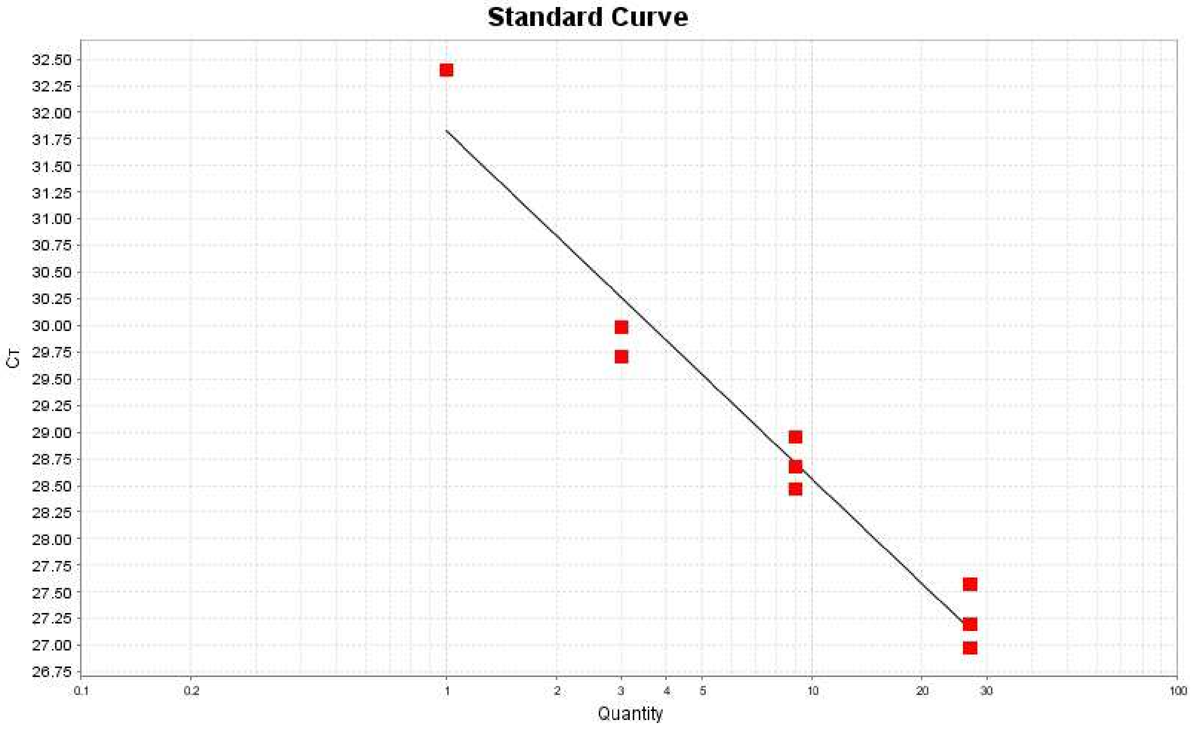

Legend
Standard
Target: EloF2 Slope:-3.267 Y-inter: 31.826 R^2: 0.953 Eff: 102%

K

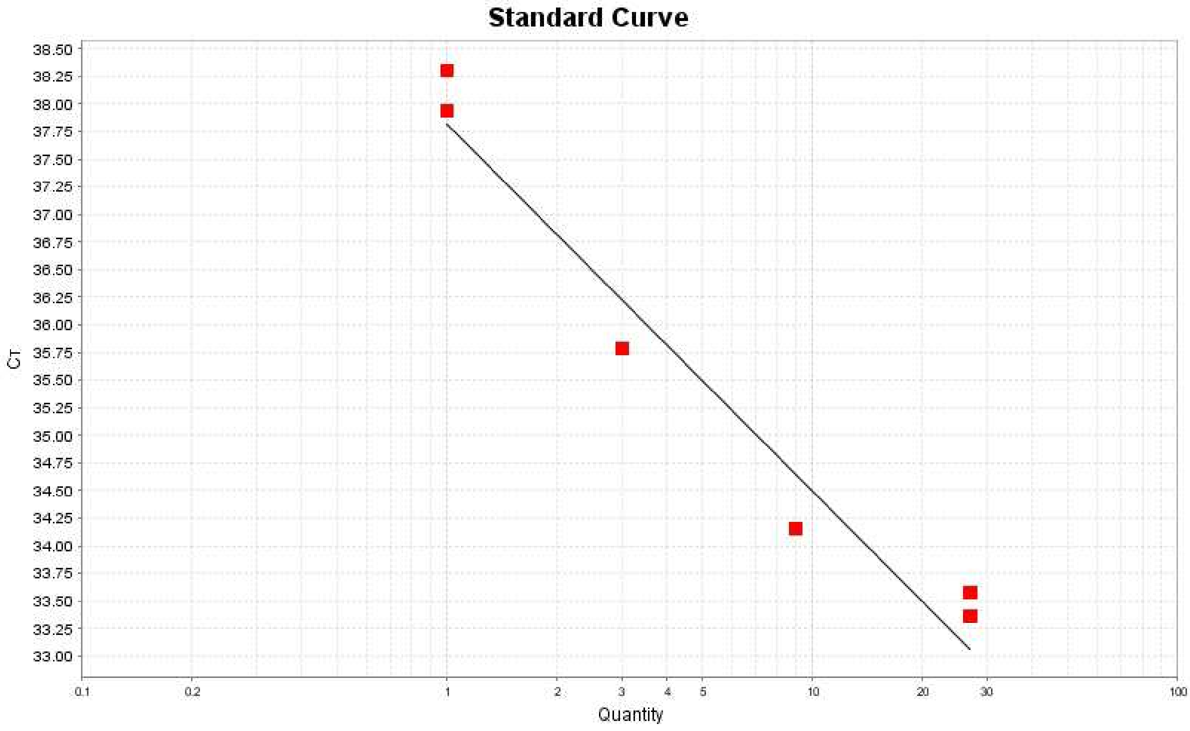

Legend
Standard

Target: PSI Slope:-3.315 Y-inter: 37.812 R^2: 0.95 Eff: 100%

L

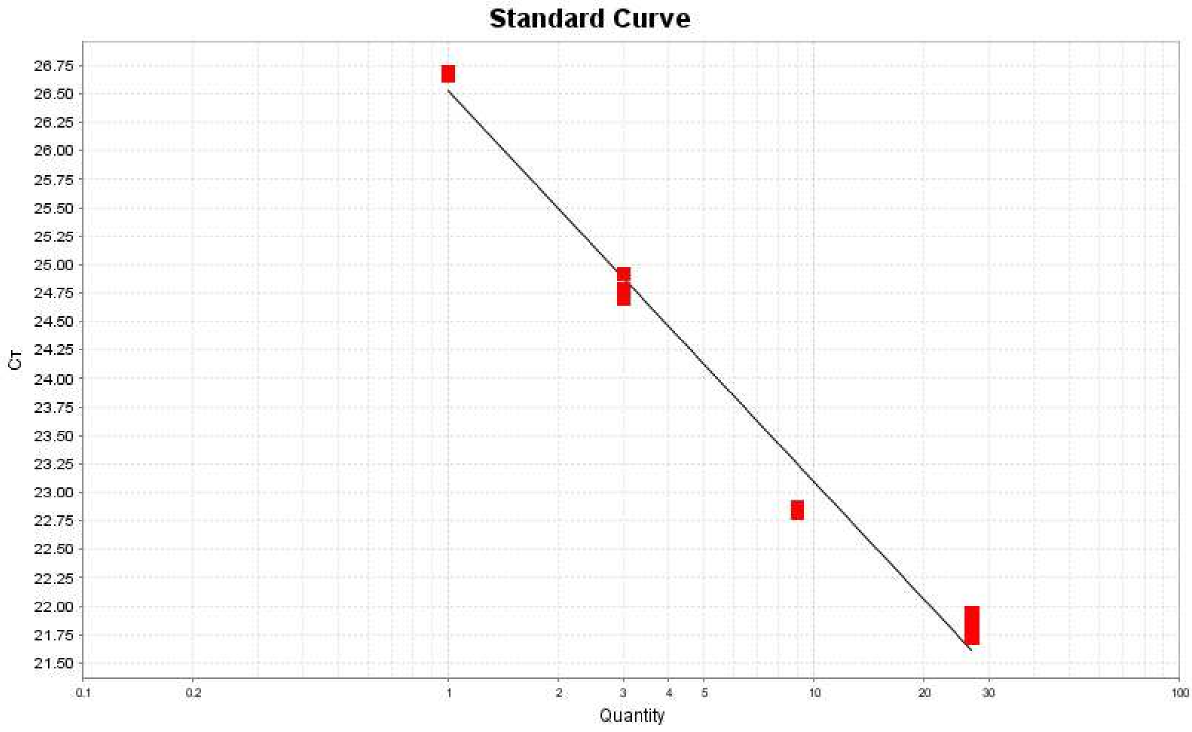

Legend
Standard

Target: HSF Slope:-3.429 Y-inter: 26.525 R^2: 0.984 Eff: 95%
